# An Active Immune Defense with a Minimal CRISPR (Clustered Regularly Interspaced Short Palindromic Repeats) RNA and without the Cas6 Protein*

**DOI:** 10.1074/jbc.M114.617506

**Published:** 2014-12-15

**Authors:** Lisa-Katharina Maier, Aris-Edda Stachler, Sita J. Saunders, Rolf Backofen, Anita Marchfelder

**Affiliations:** From the ‡ Department of Biology II, Ulm University, 89069 Ulm,; the § Bioinformatics Group, Department of Computer Science, Albert-Ludwigs-University Freiburg, Georges-Köhler-Allee 106, 79110 Freiburg, and; the ¶ BIOSS Centre for Biological Signalling Studies, Cluster of Excellence, Albert-Ludwigs-University Freiburg, Schänzlestrasse 18, 79104 Freiburg, Germany

**Keywords:** Archaea, Cas6, CRISPR/Cas, crRNA, Haloferax volcanii, Type I-B

## Abstract

The prokaryotic immune system CRISPR-Cas (clustered regularly interspaced short palindromic repeats-CRISPR-associated) is a defense system that protects prokaryotes against foreign DNA. The short CRISPR RNAs (crRNAs) are central components of this immune system. In CRISPR-Cas systems type I and III, crRNAs are generated by the endonuclease Cas6. We developed a Cas6b-independent crRNA maturation pathway for the *Haloferax* type I-B system *in vivo* that expresses a functional crRNA, which we termed independently generated crRNA (icrRNA). The icrRNA is effective in triggering degradation of an invader plasmid carrying the matching protospacer sequence. The Cas6b-independent maturation of the icrRNA allowed mutation of the repeat sequence without interfering with signals important for Cas6b processing. We generated 23 variants of the icrRNA and analyzed them for activity in the interference reaction. icrRNAs with deletions or mutations of the 3′ handle are still active in triggering an interference reaction. The complete 3′ handle could be removed without loss of activity. However, manipulations of the 5′ handle mostly led to loss of interference activity. Furthermore, we could show that in the presence of an icrRNA a strain without Cas6b (Δ*cas6b*) is still active in interference.

## Teaser

**Background:** CRISPR RNAs (crRNAs) are generated by Cas6b in type I-B systems. They are essential for the interference reaction.

**Results:** An icrRNA is generated independently from Cas6b and functions like a crRNA.

**Conclusion:** In the presence of an icrRNA, Cas6b is not required for the interference reaction.

**Significance:** This setup allows the Cas6b-independent generation of icrRNAs and thereby interference without Cas6b.

## Introduction

Prokaryotes defend themselves against invaders using several different mechanisms to degrade foreign DNA or RNA, one of which is the clustered regularly interspaced short palindromic repeat-CRISPR associated (CRISPR-Cas)^3^ system (1–6). This defense mechanism progresses in three steps as follows: 1) adaptation; 2) CRISPR RNA expression and processing; and 3) invader degradation. During the first step, the cell identifies a new invader and integrates a piece of the invader DNA (termed protospacer) into the CRISPR locus of the host (as soon as the protospacer has been integrated into the CRISPR locus, it is termed spacer). An important distinguishing characteristic for the selection of a protospacer in the type I and II CRISPR-Cas systems is the protospacer adjacent motif (PAM) (7, 8). This motif is located in the invader DNA, directly adjacent to the protospacer. The PAM sequence is important not only for its selection as a spacer but also for the third step of the process, the interference reaction. In the second step of the defense, the CRISPR RNA is synthesized yielding a long pre-crRNA that is processed into the mature functional crRNAs. These short RNAs are essential for the last step, the interference, where they detect the invader sequence and trigger degradation of the invader by Cas proteins (2, 9).

The prokaryotic immune system comes in a variety of different types that carry out the same reaction, namely the defense against foreign DNA or RNA. Data about the different systems reported show that although they carry out the same reaction they clearly differ in various aspects of the pathway. The different types of CRISPR-Cas have been grouped on the basis of their various Cas proteins into three major classes, CRISPR-Cas type I, II, and III (7), that have been further divided into initially 10 subclasses (IA–F, IIA–B, and IIIA–B) (7), with the number of newly defined subclasses constantly rising as more data about the systems are reported (10, 11).

**FIGURE 1.**
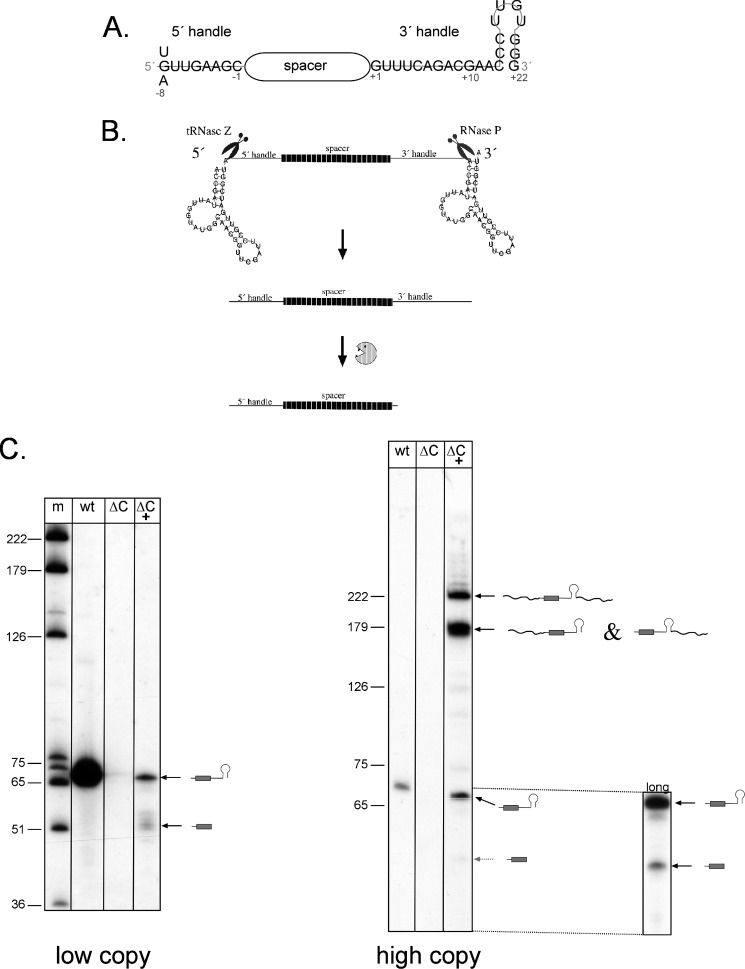
**Natural crRNA of *Haloferax* and the icrRNA.** *A,* crRNAs of *Haloferax. Haloferax* encodes three different CRISPR loci, P1, P2, and C, that have the same 30-nucleotide-long repeat sequences except for the first nucleotide of the 5′ handle (position −8 according to the nomenclature (42–44)) that is an A in P1, a U in P2, and a G in C. Thus, there are three types of crRNAs in *Haloferax* beginning with three different nucleotides. The mature crRNA contains an 8-nucleotide 5′ handle and a 22-nucleotide 3′ handle. Spacers are between 34 and 39 nucleotides long. Nucleotides in the 5′ handle are termed −8 to −1 (from the 5′ end of the 5′ handle) and nucleotides from the 3′ handle are termed +1 to +22 (42–44). *B,* maturation of the icrRNA. The pre-icrRNA contains the crRNA flanked by two t-elements. The crRNA is derived from CRISPR locus C containing spacer 1 from this locus. The t-elements are recognized and processed by RNase P and tRNase Z, generating the mature icrRNA of 66 nucleotides (icrRNA^66^). This icrRNA can be processed further to a 49-nucleotide-long icrRNA^49^ by still unknown RNases. *C,* maturation of the icrRNA in *Haloferax* cells. RNA was isolated from wild type cells (*lane wt*), *Haloferax* cells without the CRISPR locus C (*lane* Δ*C*), and ΔC cells with pTA409-telecrRNA (*lane* Δ*C* + in the *left panel*) and from ΔC cells with the high copy plasmid pTA232-telecrRNA (*lane* Δ*C* + in the *right panel*), respectively. After separation on 8% PAGE, the RNA was transferred to a membrane that was subsequently hybridized with a probe against the crRNA. The mature crRNA can be detected in wild type *Haloferax* cells but not in ΔC. *Left panel,* “*low copy,*” generation of icrRNAs from low copy plasmids. The mature icrRNA can be detected in ΔC transformed with the low copy plasmid pTA409-telecrRNA. *Lane m,* DNA size marker, sizes are given at the *left* in nucleotides. The icrRNAs are shown schematically at the *right. Right panel,* “*high copy,*” generation of icrRNAs from high copy plasmids. In *lane* Δ*C*+, the precursor of the icrRNA as well as the processing intermediates are visible. The long exposure (*bottom right,* “*long*”) shows that the shorter icrRNA of about 49 nucleotides is also present. Sizes of a DNA marker are given at the *left* in nucleotides. The precursor of the icrRNA, the intermediates, and the mature icrRNAs are shown schematically at the *right.*

The key element in all CRISPR-Cas defense systems is the crRNA. The biogenesis of the crRNA involves either two or three steps, depending on the system. In all cases, the first step is the transcription of the CRISPR RNA locus into a long precursor, the pre-crRNA. The following maturation of the crRNA is catalyzed by the Cas6 protein in CRISPR-Cas type I and type III systems. In some type I systems, Cas6 is part of the CRISPR-associated complex of antiviral defense (Cascade) (12) that consists of different Cas proteins depending on the subtype (2). In contrast, in the type III system Cas6 is a standalone endonuclease (13, 14). Processing by Cas6 within the repeat sequence directly yields the mature functional crRNA in types I-A, I-E, and I-F (9). The resulting crRNA consists of an eight-nucleotide repeat-derived 5′ handle, the invader-targeting spacer sequence, and the 3′ handle, which contains the remainder of the repeat sequence (Fig. 1*A*) (2). In some type I systems (I-E and I-F), the Cas6 proteins stay bound to the crRNA after processing. In type III systems, a second maturation step is observed after Cas6 processing, which shortens the crRNA 3′ end and sometimes removes the complete repeat sequence downstream of the spacer (14, 15).

The initial invader DNA recognition is governed by Watson-Crick base pairing with a 7–10-nt segment of the crRNA referred to as the “seed” sequence (16–19). The seed sequence is involved in initial pairing between crRNA and invader, and it allows rapid probing of different regions of cellular nucleic acids. If a perfect match between seed sequence and target DNA is found, the remainder of the spacer sequence of the crRNA base pairs with the invader DNA. In the type I-E system, the seed sequence is a seven-nucleotide-long noncontiguous sequence between the 5′ end of the crRNA spacer sequence and the invader (17). In the type I-B system, this seed sequence is slightly longer with 10 nucleotides (20). An additional prerequisite for the interference is the presence of the PAM sequence in the invader DNA (2).

Here, we investigate the function of Cas6 in the interference reaction and the essential requirements for the crRNA in the type I-B system of the archaeon *Haloferax volcanii. H. volcanii* contains only one CRISPR-Cas system (I-B) that consists of eight Cas proteins (Cas1 to Cas5, Cas6b,^4^ Cas7, and Cas8b) and three CRISPR RNA arrays (20). We could previously identify the PAM sequences for this system showing that six different PAMs are active in triggering degradation (21). The *Haloferax* I-B system has a Cascade-like complex, with Cas6b copurifying with the Cas5 and Cas7 proteins and the crRNA (22). It has been shown that the Cas6b protein is involved in crRNA maturation and that the crRNA 5′ handles are eight nucleotides long; however, different 3′ lengths have been reported (22).

We developed here a Cas6b-independent crRNA maturation pathway for the *Haloferax* type I-B system *in vivo* that expresses a functional crRNA, which we termed independently generated crRNA (icrRNA). The icrRNA is transcribed with flanking tRNA-like structures (so-called t-elements) that are processed by the tRNA processing enzymes RNase P and tRNase Z (23). The icrRNA is effective in triggering degradation of an invader plasmid carrying the matching protospacer sequence.

We show here that a minimal crRNA in the I-B system needs a seven-nucleotide 5′ handle and does not require a 3′ handle at all. In addition, we show that the Cas6b protein is not required for the interference reaction when an icrRNA is present. With the Cas6b-independent maturation pathway developed here, the first *in vivo* analysis of crRNA characteristics essential for the interference reaction was possible.

## EXPERIMENTAL PROCEDURES

### 

#### 

##### Strains

*H. volcanii* strains H119 (strains used are listed in Table 1), Δ*cas6* (Δ*pyrE2*, Δ*leuB*, Δ*trpA*, and Δ*cas6*) (22), and Δ*C* (Δ*pyrE2*, Δ*leuB*, Δ*trpA*, and HVO_2,385,045–2,386,660::trpA) (this study) were grown aerobically at 45 °C in Hv-YPC medium (21). *H. volcanii* strains Δ*cas6* and Δ*C* containing plasmids were grown in Hv-Ca or Hv-min medium with the appropriate supplements. *Escherichia coli* strains DH5α (Invitrogen) and GM121 (24) were grown aerobically at 37 °C in 2YT medium (25).

**TABLE 1:** **Strains, plasmids, and primers used in this study**

**Strains**		
DH5α	F− ϕ80*lac*ZΔM15 Δ(*lac*ZYA-*arg*F) U169 *rec*A1 *end*A1 *hsd*R17 (rk-, mk+) *gal*- *pho*A *sup*E44 λ- *thi*-1 *gyr*A96 *rel*A1	Invitrogen
GM121	F− *dam-3 dcm-6 ara-14 fhuA31 galK2 galT22 hdsR3 lacY1 leu-6 thi-1 thr-1 tsx-78*	24
H119	Δ*pyrE2* ΔtrpA Δ*leu*B	27
Δ*cas6*	Δ*pyrE2*, Δ*leuB*, Δ*trpA*, Δ*cas6*	22
Δ*C*	Δ*pyrE2*, Δ*leuB*, Δ*trpA*, ΔHVO_2,385,045–2,386,660::trpA	This study

**Plasmids**		
pTA409	Shuttle vector with *pyrE2* marker and pHV1 replication origin	26
pTA352	Shuttle vector with *leu*B marker and pHV1 replication origin	31
pTA409- PAM3CSp1	Spacer C1 downstream of PAM3 (TTC)	16
pTA352- PAM3CSp1	Spacer C1 downstream of PAM3 (TTC)	30
pMA-RQ-telecrRNA	*E. coli* plasmid containing the promoter, crRNA flanked by t-elements and terminator, expressing the icrRNA	This study
pMA-telecrRNA	*E. coli* plasmid containing the promoter, crRNA flanked by t-elements and terminator, expressing the icrRNA	This study
pTA409-telecrRNA	Plasmid containing the promoter, crRNA flanked by t-elements and terminator, expressing the icrRNA	This study
pTA232-telecrRNA	Plasmid containing the promoter, crRNA flanked by t-elements and terminator, expressing the icrRNA	This study
pTA232-telecrRNAX	Like pTA232-telecrRNA but containing telecrRNA mutants (X = 1–23)	This study
pTA131Cup	Upstream region of CRISPR RNA gene locus C	This study
pTA131-Cupdo	Up- and downstream regions of CRISPR RNA gene locus C	This study
pTA131-CupdoTrp	Up- and downstream regions of CRISPR RNA gene locus C flanking the *trpA* marker gene	This study

**Primers**		
itele1	ACCGATATTGGTATGGCAACC	This study
del1	AAGGGTTCGTCTGAAACTTTCTG	This study
del2	TTCGTCTGAAACTTTCTGAGATTC	This study
del3	CTGAAACTTTCTGAGATTCGAGG	This study
del4	ACTTTCTGAGATTCGAGGGCATC	This study
C-SP1	CTGAGATTCGAGGGCATCTTCGGACCTTTCC	This study
DOmitteC	GAGAAGCTTAAATACAACCA	This study
Cdelup	TATAGGTACCCGCTCGTCGGTGAGTCGCTCACCGACTTCCG	This study
Cdelupi	TATAGATATCCGAGGCGGAGCGTCGAGAGCGCTAGTC	This study
Cdeldo	TATATCTAGACGTGCGAGAACTCGTCGACGGACTCGTCC	This study
Cdeldoi	TATAGATATCCGAAGTGAAGAATCAGGAGACGGCATTGC	This study

##### Construction of Plasmids and Transformation of H. volcanii

The plasmids for expressing icrRNA (pTA409-telecrRNA, pTA232-telecrRNA, and telecrRNA variants in both vectors) were generated as follows (plasmids are listed in Table 1). The DNA fragment containing the crRNA or crRNA mutants flanked by t-elements were ordered from GeneArt® as plasmids pMA-RQ-telecrRNA and pMA-telecrRNA. Plasmids contained a synthetic *Haloferax* promoter,^5^ the crRNA, flanked by t-elements and a synthetic *Haloferax* terminator.^5^ Plasmids were digested with KpnI and BamHI to isolate the DNA fragment containing the complete insert. The resulting fragment was cloned into pTA409 (26) and pTA232 (27) (both digested with KpnI and BamHI). Four crRNA mutants were generated by inverse PCR on pMA-telecrRNA using primer pairs (primer sequences are listed in Table 1) itele1/del1, itele1/del1, itele1/del1, and itele1/del1 to generate variant 13 (deletion of the last five nucleotides of the 3′ handle), 14 (deletion of the last 10 nucleotides of the 3′ handle), 15 (deletion of the last 15 nucleotides of the 3′ handle), and 16 (deletion of the last 20 nucleotides of the 3′ handle), respectively. In preparation for transformation, all plasmids were passaged through *E. coli* GM121 cells to avoid methylation. *Haloferax* cells were subsequently transformed using the polyethylene glycol method (27, 28).

##### Generation of a CRISPR Locus C Gene Deletion Strain (ΔC)

The deletion of the CRISPR locus C was achieved by using the pop-in/pop-out method as described previously (24, 25, 29). The region upstream of the gene for CRISPR locus C was PCR-amplified with flanking regions from the chromosomal DNA of *H. volcanii* strain H119 using primers Cdelup (containing the restriction site KpnI) and Cdelupi (containing the restriction site EcoRV). The resulting 300-bp PCR fragment was subsequently cloned into the vector pTA131 (digested with KpnI and EcoRV), yielding pTA131-Cup. Next, the region downstream of the locus C gene was amplified using primers Cdeldo (containing the restriction site XbaI) and Cdeldoi (containing the restriction site EcoRV). The resulting 500-bp fragment was cloned into the plasmid pTA131-Cup (digested with EcoRV and XbaI), yielding plasmid pTA131-Cupdo. This plasmid was digested with EcoRV to insert the marker gene *trpA* (coding for tryptophan synthase A). The tryptophan marker *trpA* was amplified using plasmid pTA132 (27) as template and oligonucleotides TRP1/TRP2, and cloning of the *trpA* marker gene into the plasmid pTA131-Cupdo resulted in pTA131-CupdoTrp. Plasmids were passaged through *E. coli* GM121 to prevent methylation, and *H. volcanii* strain H119 was subsequently transformed with this construct to allow integration (pop-in) of the plasmid into the genome. The subsequent selection for loss of the *pyrE2* marker by plating on 5-fluoroorotic acid revealed pop-out mutants. To confirm the removal of the gene for CRISPR locus C, we performed a Southern blot analysis. Chromosomal DNA was isolated from the wild type and potential locus C gene deletion mutants. Southern blot hybridization was performed as described (27), with the following modifications. 10 μg of SacII-digested DNA was separated on a 0.8% agarose gel and transferred to a nylon membrane (Hybond^TM^-N, GE Healthcare). A 250-bp fragment of the downstream region of locus C was amplified using primers Cdeldoi and DOmitteC, and the fragment was radioactively labeled using [α-^32^P]dCTP and random prime kit Readiprime^TM^II (GE Healthcare) and subsequently used as a hybridization probe.

##### Plasmid Invader Tests

The invader plasmid constructs pTA352-PAM3CSp1 (30) and pTA409-PAM3CSp1 (16) were generated based on the *Haloferax* shuttle vectors pTA352 (pHV1, *leuB*) (31) and pTA409 (pHV1, *pyrE2*) (26), including spacer 1 of the CRISPR locus C (C1) and the PAM sequence TTC (PAM3) (16, 21). As a control reaction, *Haloferax* cells expressing the icrRNA (WT or mutants) were transformed with the vector without insert (pTA352 or pTA409). Plasmids were passaged through *E. coli* GM121 cells (to avoid methylation) and were then introduced into *Haloferax* cells using the PEG method (27, 28). To confirm the identification of a functional invader sequence, *H. volcanii* cells were transformed at least three times with the plasmid invader construct or the control vector. For plasmid invader tests, transformations with at least a 100-fold reduction in transformation rates are considered successful interference reactions (21, 32). High reductions in transformation rates provide evidence for high targeting efficiency of the crRNA analyzed.

##### Northern Blot Hybridization

Total RNA was isolated, unless stated otherwise, from exponentially growing *H. volcanii* cells as described (16). After separation of 10 μg of RNA (total RNA) on 8% denaturing gels, RNA molecules were transferred to nylon membranes (Hybond-N+, GE Healthcare) and incubated with oligonucleotides against the spacer 1 from locus C (primer C1). The primer was radioactively labeled at the 5′ end with [γ-^32^P]ATP and subsequently used for hybridization.

##### Investigation of icrRNAs

To determine the exact length and sequence of the crRNA, RNA was isolated from wild type *Haloferax* cells (H119) and strain ΔC × pTA232-telecrRNA grown to an absorbance of 0.74. RNA was separated on 8% PAGE, and RNA ranging in size from 45 to 55 nucleotides (fraction 1) and from 60 to 75 nucleotides (fraction 2) was eluted and sent to vertis Biotechnologie AG for cDNA preparation and RNAseq analysis. The RNA samples were first treated with polynucleotide kinase and then poly(A)-tailed using poly(A) polymerase. Afterward, an RNA adapter was ligated to the 5′-monophosphate of the RNA. First-strand cDNA synthesis was performed using an oligo(dT)-adapter primer and the Moloney murine leukemia virus reverse transcriptase. The resulting cDNAs were PCR-amplified to about 10–30 ng/μl using a high fidelity DNA polymerase. The cDNAs were purified using the Agencourt AMPure XP kit (Beckman Coulter Genomics) and were analyzed by capillary electrophoresis. For Illumina sequencing, the cDNA samples were mixed in approximately equal amounts. An aliquot of the cDNA pool was analyzed by capillary electrophoresis. The primers used for PCR amplification were designed for TruSeq sequencing according to the instructions of Illumina.

##### RNAseq Mapping

First, original reads were trimmed according to their sequencing quality using the fastq_quality_trimmer program from the FASTX-Toolkit version 0.0.13 with the options “-t 13-Q 33.” The parameter Q is required due to the ASCII offset of 33 used for the quality scores in the Sanger format. The estimated probability that a base call is incorrect (*p* > 0.05) corresponds to quality values below 13 (33). Second, trimmed reads were mapped with Segemehl (34) version 0.1.3 with the options “–polyA –prime3′AGATCGGAAGAGCGTCGTGTAGGGAAAGAGTGTAGATCTCGGTGGTCGCCGTATCATT′” .

This setting removes the poly(A) tail and the 3′ Illumina sequencing adapter. The following percentages of the original reads were successfully mapped from each sample: 86% for S1 (wild type RNA fraction of 60–75 nt length), 74% for S2 (wild type RNA fraction of 45–55 nt length), 91% for S3 (icrRNA fraction of 60–75 nt length), and 81% for S4 (icrRNA fraction of 45–55 nt length). All samples had 20–40 million reads. Subsequent to mapping, alignments were filtered such that they had a maximum edit distance of 2, were located on the reverse strand (because CRISPR locus C is transcribed from the reverse strand), and matched uniquely to the genome. The filtering produced a clearer signal, but it did not change original profiles. To explore and display RNAseq results, we used the Integrative Genomics Viewer version 2.0.3 (35).

## RESULTS

To determine the essential nucleotides of the crRNA for the interference and to investigate whether the Cas6b protein is required for the interference reaction, we established a Cas6b-independent crRNA generation in *H. volcanii*. Using this setup, we could study the effect of crRNA mutants on the interference reaction independently of the crRNA processing stage; thus, we captured crRNA characteristics that were specific to the interference reaction.

### 

#### 

##### Cas Protein-independent Generation of crRNAs

We generated a plasmid that encodes the crRNA as well as t-elements directly up- and downstream of the crRNA (Fig. 1*B*). The crRNA is derived from the *Haloferax* CRISPR locus C and contains spacer 1 of this locus. The t-element is a tRNA-like structure that has been previously detected directly upstream of the 5 S rRNA in *H. volcanii*, and it is processed by tRNase Z to generate the 5 S rRNA 5′ end (36, 37). Generally t-elements are substrates for both tRNA-processing enzymes, the 5′-processing enzyme RNase P, and the 3′-processing enzyme tRNase Z (36, 38). Processing of the t-elements up- and downstream of the crRNA should yield the mature icrRNA. We cloned the crRNA/t-element insert into the *Haloferax* vector pTA409 (26), yielding pTA409-telecrRNA. A *Haloferax* strain that has the CRISPR locus C deleted (strain ΔC) was generated to get a strain without the endogenous spacer 1 from locus C (see under “Experimental Procedures”). This strain was transformed with plasmid pTA409-telecrRNA yielding ΔC × pTA409-telecrRNA. Northern blot analysis showed that an icrRNA is generated with the same size as the crRNA made in the wild type strain (which generates the crRNA from the CRISPR locus C) (Fig. 1*C*). Thus, the icrRNA is efficiently generated from the plasmid. In addition, some shorter RNAs are visible, and these shorter crRNAs have also been reported earlier in wild type cells (22). Because the amount of icrRNA was rather low compared with the endogenous crRNA, we cloned the crRNA/t-element insert into a *Haloferax* vector with a higher copy number, pTA232 (27), yielding pTA232-telecrRNA. Northern analysis showed that a *Haloferax* ΔC strain transformed with pTA232-telecrRNA indeed generated higher amounts of icrRNA (Fig. 1*C*).

**FIGURE 2.**
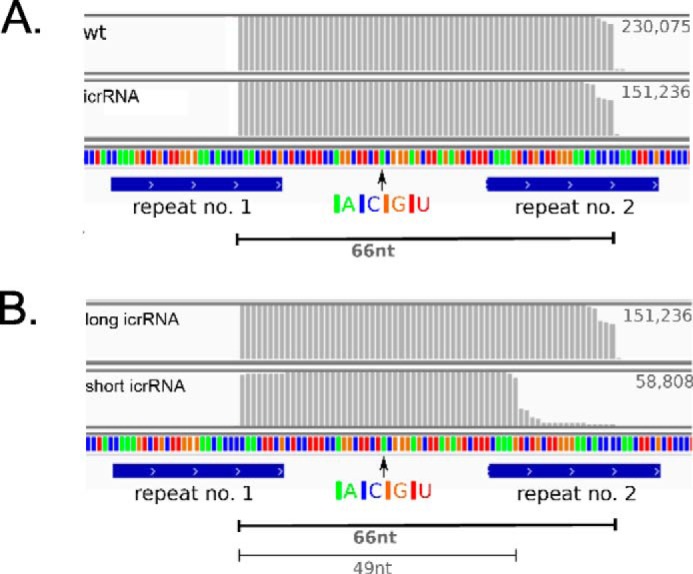
**Determination of crRNA and icrRNA sequences with RNAseq.** *A,* comparison of Cas6b catalyzed crRNA generation (*wt*) and Cas6b independent crRNA production (icrRNA). RNAseq data from RNA fractions (sizes 60–75 nucleotides) isolated from wild type *Haloferax* cells (*upper row* “*wt*”) and ΔC × pTA232-telecrRNA (*lower row* “*icrRNA*”) were mapped to the CRISPR C locus. The icrRNA only comprises spacer 1, between repeats 1 and 2. The *numbers* to the right of each row reflect the number of reads mapping to this region. The dominant crRNA length is 66 nt, and each mature crRNA begins with the characteristic eight nucleotide handle at its 5′ end and ends with the remaining 22 nucleotides of the repeat. Both pathways produce the same mature crRNA. *B,* two types of icrRNA are generated. In ΔC × pTA232-telecrRNA, in addition to the 66-nucleotide-long icrRNA, a shorter icrRNA of 49 nt is also evident (Fig. 1*C*). RNAseq data from the longer icrRNA fraction (sizes 60–75 nucleotides) isolated from ΔC × pTA232-telecrRNA *Haloferax* cells (*upper row,* “*long icrRNA*”) and from the shorter icrRNA fraction (sizes 45–55 nucleotides) (*lower row*, “*short icrRNA*”) were mapped to the CRISPR C locus. Each icrRNA begins with the characteristic eight-nucleotide 5′ handle, followed by the spacer sequence. In contrast to the long crRNAs, the shorter crRNAs contain only a five-nucleotide long 3′ handle.

To confirm that processing of the icrRNA yielded exactly the same 5′ and 3′ ends as in the “natural” crRNA production, we isolated the two RNA fractions that contained the long crRNA of about 65 nucleotides (RNA fraction of 60–75 nucleotides in length isolated) and the shorter crRNA of about 51 nucleotides (RNA fraction of 45–55 nucleotides in length isolated) from wild type *Haloferax* cells and ΔC × pTA232-telecrRNA cells and analyzed them with RNAseq. The icrRNAs from the 60–75-nucleotide fraction (isolated from ΔC × pTA232-telecrRNA strain) have exactly the same 5′ and 3′ ends as the wild type crRNA (Fig. 2*A*). Thus, we could show that we can generate a mature icrRNA identical to the natural crRNA in *Haloferax* cells. In addition we could show that a slightly shorter icrRNA version with 49 nucleotides in length (icrRNA^49^) is also present (Fig. 2*B*). This shorter icrRNA^49^ has the same 5′ end but a 17-nucleotide shorter 3′ handle than icrRNA^66^.

The only difference between the natural crRNA and the icrRNAs is the nature of the processing product end groups; the icrRNA contains a 5′-phosphate group at the crRNA 5′ end and a 3′ hydroxyl group at the crRNA 3′ end due to processing by RNase P and tRNase Z (23, 39). This is in contrast to the observed end groups generated naturally by type I Cas6 processing as follows: a 5′ hydroxyl group and 2′–3′ cyclic phosphate (I-C and I-E) (13, 40, 41) or a noncyclic 3′ phosphate (I-F) (18). However, we show here that the nature of the end group is not important for the interference reaction (see below). Taken together, we could successfully establish a Cas6b-independent crRNA maturation pathway.

##### icrRNAs Are Active in Interference

To investigate whether the icrRNA is active in interference, we challenged *Haloferax* strain ΔC expressing the icrRNA (from the high copy plasmid pTA232-telecrRNA) with an invader plasmid (21). The invader plasmid contains the protospacer sequence that matches spacer 1 of CRISPR locus C from *Haloferax*; thus, this sequence can be detected by the icrRNA. Adjacent to the protospacer is the PAM sequence TTC that is one of the six PAMs shown to be active in *Haloferax* to trigger degradation (21). If this invader plasmid is recognized as an invader, it is degraded by the defense system, and cells cannot grow on selective medium. Transformation rates of strains transformed with the invader were reduced more than 100-fold compared with transformation with a control plasmid, showing that the invader plasmid is recognized and degraded (Table 2). The same experiment was subsequently carried out with the low copy icrRNA plasmid (pTA409-telecrRNA). Again the transformation rates were reduced in comparison with a control plasmid, showing that the lower levels of icrRNA can also trigger the interference reaction. Taken together, the icrRNAs can trigger the interference reaction and thus are fully functional crRNAs.

**TABLE 2:** **Interference test with the icrRNA** Targeting efficiencies of the icrRNAs expressed from the high copy and low copy icrRNA plasmids were analyzed. The targeting efficiency of the icrRNAs expressed from the high copy icrRNA plasmid were investigated in strain Δ*C* and Δ*cas6b*. A successful interference reaction reduces the transformation rate by at least a factor of 0.01, demonstrating a high targeting efficiency of the icrRNA (21). If the plasmid is not recognized as an invader and is not destroyed, the transformation rate is the same as with a normal plasmid; there is no reduction of transformation rate. If the plasmid is recognized as an invader and degraded, cells cannot survive on ura^−^ medium. However, some cells can inactivate the CRISPR-Cas system (by deleting or mutating the *cas* genes or the genes for the CRISPR RNAs) and can grow on the selective medium (21). As a result, the plates are not completely empty since the mutated *Haloferax* cells can grow. Therefore, a high targeting efficiency is defined by a reduction in transformation rate by at least 0.01 (21).

Strain	icrRNA plasmid	Reduction in transformation rate by factor
Δ*C*	pTA409-telecrRNA (low copy)	0.01
Δ*C*	pTA232-telecrRNA (high copy)	0.01
Δ*cas6b*	pTA232-telecrRNA (high copy)	0.0006

##### Cas6b Is Not Required for Interference in the Presence of icrRNAs

In the wild type situation Cas6b is required for crRNA production, and it is conceivable that it could also be required for the interference reaction, because it was shown to be part of Cascade in *Haloferax* (I-B system), *E. coli* (I-E), *Pseudomonas aeruginosa* (I-F), and *Sulfolobus solfataricus* (I-A) (18, 22, 42–46). By the Cas6b-independent generation of icrRNAs, we separated the role of Cas6b in crRNA processing from its function in the interference reaction. Using icrRNAs, we can now determine whether Cas6b is also important for the interference reaction. Thus, we transformed a Δ*cas6b* strain with pTA232-telecrRNA and subsequently with the invader plasmid. The transformation rate of these cells was greatly reduced (by factor 0.0006) (Table 2), showing that the interference reaction works without Cas6b. In the Δ*cas6b* strain, no internal crRNAs can be generated; thus, the only crRNAs present in these cells are the icrRNAs. Subsequently Cascade complexes can only be loaded with icrRNAs. This might explain the greater reduction in the transformation rate compared with Δ*C*; all Cascades in Δ*cas6b* contain the icrRNA directed against the invader plasmid, whereas in ΔC only a percentage of the Cascade complexes are loaded with an icrRNA because the crRNAs from CRISPR locus P1 and P2 are also present. Taken together, the Cas6b protein is not required for the interference reaction when the icrRNA is present.

##### Essential Features of the crRNA 5′ Handle

**FIGURE 3.**
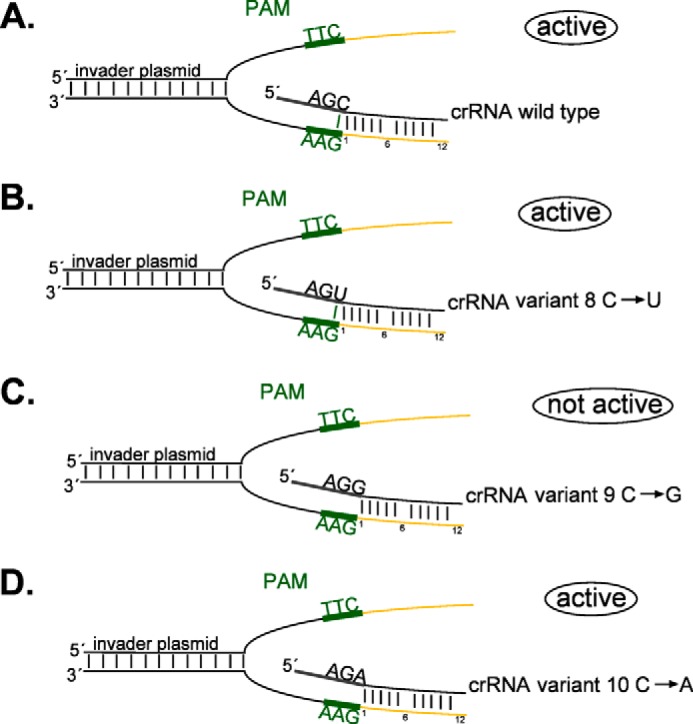
**Interaction of the crRNA with the complementary PAM sequence.** Details of the interaction between crRNA and invader plasmid DNA are shown. The spacer sequence of the crRNA base pairs with the protospacer sequence (except for every sixth nucleotide). The PAM sequence and its complementary sequence are shown in *green*. The protospacer sequence is shown in *yellow*. The last three nucleotides (−3 to −1) of the crRNA are shown. The −1 crRNA nucleotide is located directly opposite the third complementary PAM nucleotide. *A,* in the wild type crRNA, the −1 nucleotide is a C that can base pair with the third complementary PAM nucleotide G. *B,* in the crRNA variant 8, the −1 nucleotide is a U that can base pair with the complementary PAM nucleotide G. *C,* in variant 9 the −1 nucleotide is a G that cannot base pair with G. *D,* in variant 10, the −1 nucleotide is an A that cannot base pair with the third complementary PAM nucleotide G. The functionality of each crRNA is indicated with “active” or “not active”.

Because the icrRNA was proven to be identical to the “naturally” expressed crRNA and to be fully active in interference, we generated different versions of the icrRNA to analyze the essential features of a crRNA for the interference reaction. To identify the important nucleotides of the 5′ handle, we generated 10 different variants and analyzed them for activity in the interference reaction (Table 3). All variants were transformed into strain Δ*cas6b* that was subsequently challenged with the invader plasmid. First, we mutated the first nucleotide of the crRNA (which is a G) to a A, U, or C (variants 4–6). Mutation of the first nucleotide (position −8) results in icrRNAs that are as effective in interference as the wild type icrRNA (Table 3). This is in agreement with the *in vivo* situation in *Haloferax*, where the crRNAs are generated from three different CRISPR loci, each of which have a different nucleotide at position −8 of the 5′ handle (Fig. 1*A*). Second, the −1 nucleotide was mutated from C to U and G and A (variants 8–10). This nucleotide has been shown in *E. coli* (type I-E) to be derived from the invader (47–49). In *Haloferax,* the nucleotide −1 is a C and thus also identical to the last nucleotide of the PAM used in this study (TTC). It therefore has the potential to base pair with the invader (Fig. 3*A*). Mutation of this nucleotide to a U does not interfere with the defense activity. The U at this position could still base pair with the complementary PAM sequence in the invader (U-G base pair) (Fig. 3*B*). Mutation of the −1 nucleotide to a G, however, abolishes the defense activity, and this nucleotide could not base pair any longer with the complementary PAM sequence (G*X*G) (Fig. 3*C*). Surprisingly, mutation of this nucleotide from C to an A does not interfere with the defense activity, although an A at this position is not able to base pair with the complementary PAM sequence in the invader (G*X*A) (Fig. 3*D*).

**TABLE 3:**
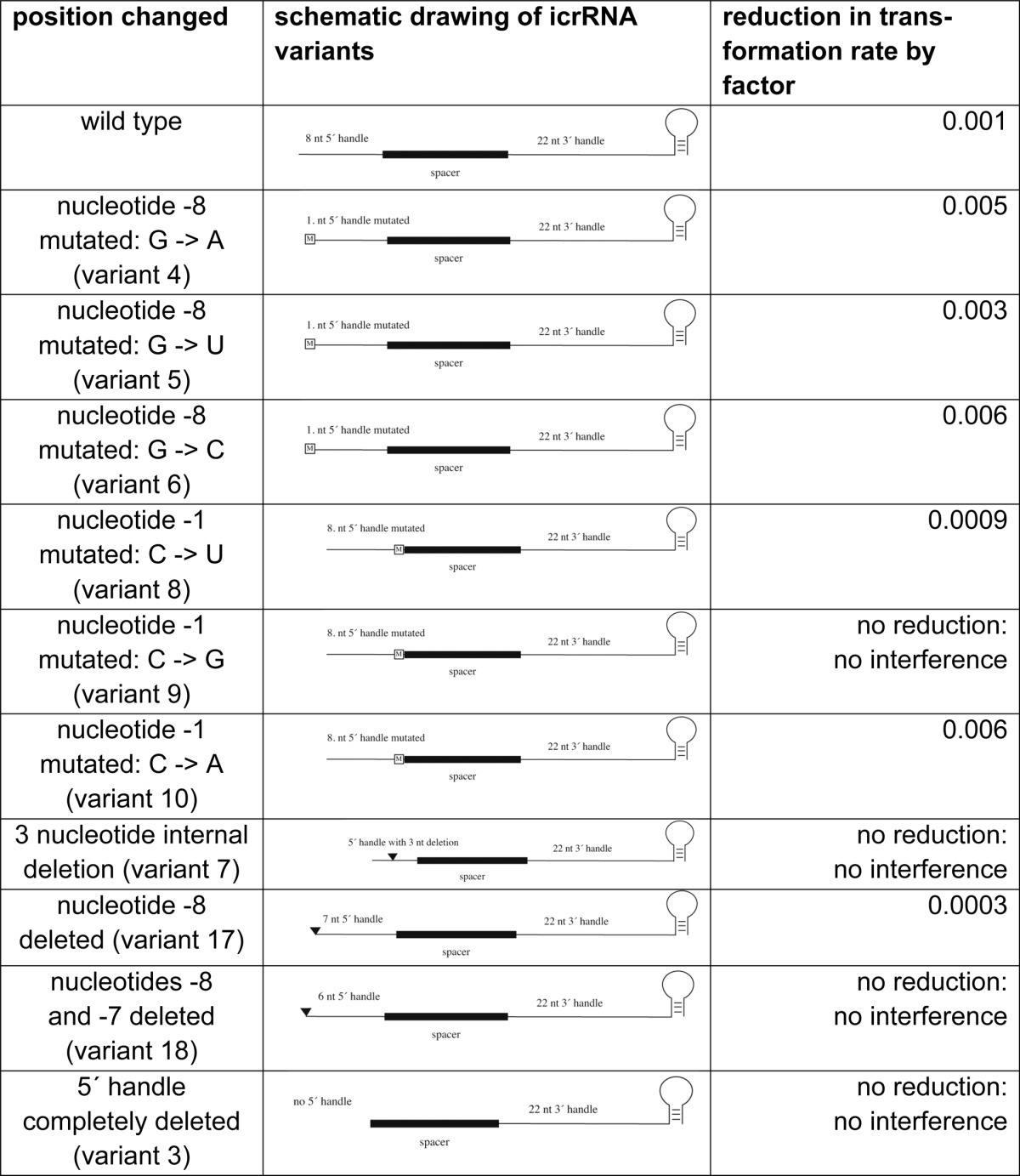
**crRNA 5′ handle is essential** Ten different variants of the icrRNA with different mutations in the 5′ handle were generated. The reduction of transformation rates upon transformation of ΔC × pTA232-telecrRNA with invader plasmid is shown (see column Reduction of transformation rate by factor), demonstrating the targeting efficiency of the icrRNA variants. A successful interference reaction reduces the transformation rate by at least a factor of 0.01 (21). If the plasmid is not recognized as an invader and is not destroyed, the transformation rate is the same as with a normal plasmid, and there is no reduction of transformation rate.

Because the nature of the first crRNA nucleotide is not important, we next deleted this nucleotide, generating an icrRNA that is still active in interference. Deletion of the first two nucleotides results however in an icrRNA inactive in interference. A deletion of three nucleotides in the 5′ handle (positions −6 to −4) (variant 7) is not tolerated. The complete removal of the 5′ handle (variant 3) results in a crRNA that cannot trigger the interference reaction anymore.

Taken together, mutations in the 5′ handle are tolerated at the first nucleotide (position −8) and to some extent at position −1. Only the deletion of the first nucleotide of the 5′ handle is tolerated, and all other deletions result in inactive icrRNAs.

##### Essential Features of the crRNA 3′ Handle

The crRNA 3′ handle in *Haloferax* has the potential to form a short stem loop structure at the very 3′ end (Fig. 1*A*). To determine whether parts of this stem loop are required and to define the essential features of the 3′ handle, we constructed 13 icrRNA variants with mutations in the 3′ handle and analyzed their activity in interference (Table 4). We mutated a nucleotide in the loop of the potential stem loop structure (G to C or U) (variants 11 and 12). These variants were both still active in triggering the interference reaction. The removal of four nucleotides of the 3′ handle in variant 1 (positions 8–11 in the 3′ handle) also did not interfere with the interference reaction. Likewise, the removal of 11 nucleotides in variant 2 (positions 1–11) did not reduce the interference. The nature of the 3′ handle differs from CRISPR system to CRISPR system. In *Haloferax* wild type cells, two types of crRNAs are observed having a 3′ handle of ∼22 nucleotides and ∼5 nucleotides (22). A similar observation was made with the icrRNA, because a long and a short icrRNA can be detected (Figs. 1*C* and 2*B*) that contains a 22-nucleotide and a 5 nucleotide 3′ handle (Fig. 2*B*). To investigate how many nucleotides can be removed from the 3′ handle, we designed several 3′ handle deletion variants. The five terminal nucleotides were deleted in variant 13; 10 terminal nucleotides were removed in variant 14, and the last 15 and 20 nucleotides were deleted in variants 15 and 16, respectively. The interference tests clearly show that all four deletions in the 3′ handle had no effect on the interference activity (Table 4). In variant 20, only one nucleotide of the 3′ handle remained, but still this crRNA was effective in triggering the interference reaction. This last nucleotide was mutated in variants 21–23 from a G to a C, A, or U. Again, all variants were still active. Even a complete removal of all 22 nucleotides (variant 19) did not interfere with the interference reaction. These results also suggest that the exact length of the complete crRNA is not important, because different lengths at the 3′ handle are tolerated.

**TABLE 4:**
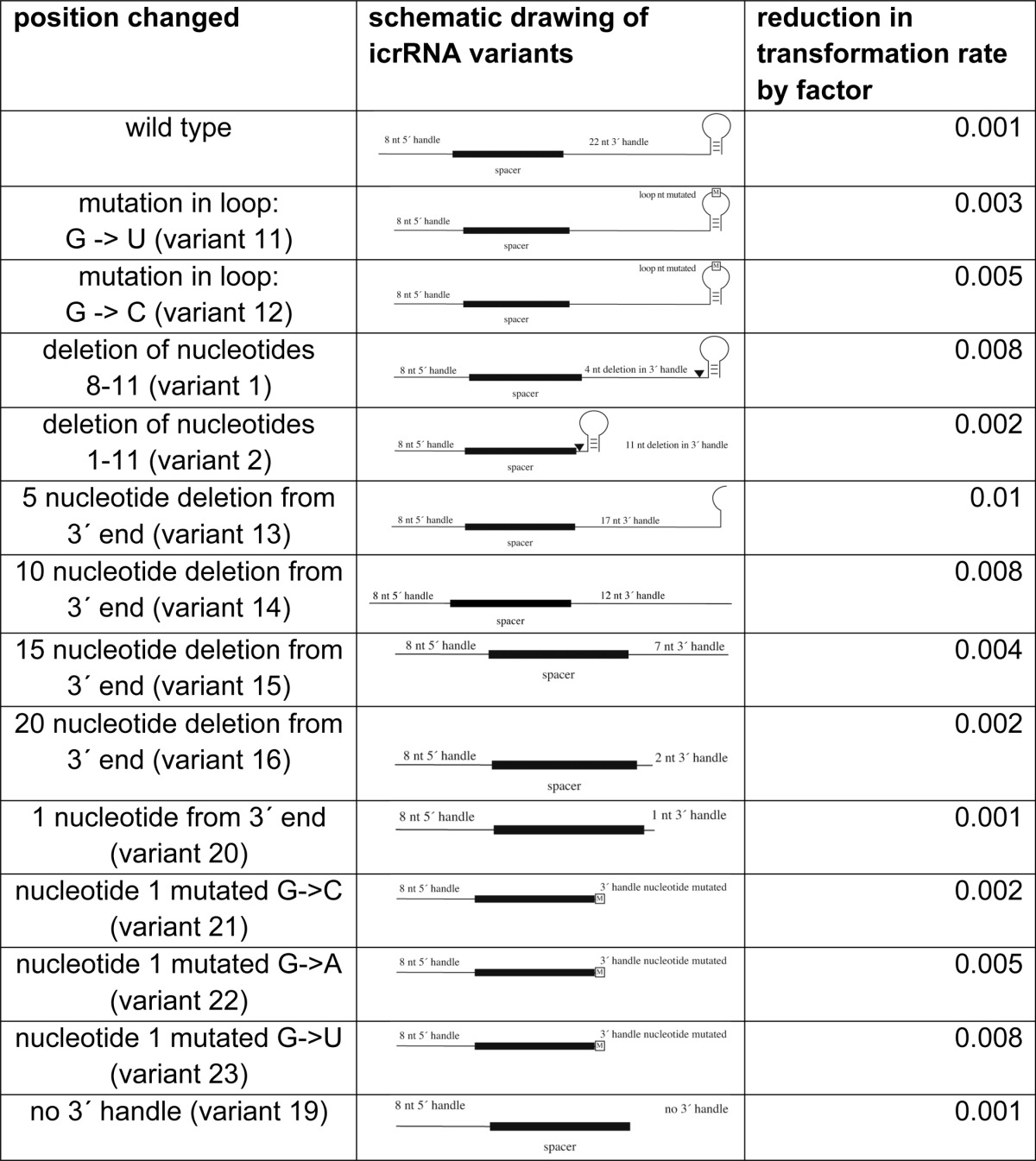
**crRNA 3′ handle can be omitted** Thirteen different variants of the icrRNA with different mutations in the 3′ handle were generated. The reduction of transformation rates upon transformation with invader plasmids is shown (see column Reduction of transformation rate by factor), demonstrating the targeting efficiency of the icrRNA variants. A successful interference reaction reduces the transformation rate by at least factor 0.01 (21). If the plasmid is not recognized as an invader and is not destroyed, the transformation rate is the same as with a normal plasmid, and there is no reduction of transformation rate.

## DISCUSSION

We could successfully establish a Cas6b-independent crRNA maturation pathway in *Haloferax* cells. In this pathway, icrRNAs are excised from a precursor with the help of tRNA processing enzymes, resulting in small RNAs active in the interference reaction. The icrRNAs are identical to the natural crRNAs except for the nature of the end groups.

### 

#### 

##### Cas6b Is Only Required for crRNA Maturation in Type I-B

Using the independently generated crRNA, we could show that Cas6b is not required for any other reactions besides crRNA processing in the prokaryotic immune system I-B. As soon as the crRNA is generated without Cas6b, this protein is dispensable, because it is not required for the interference reaction. We previously showed that Cas6b copurifies with Cascade in *Haloferax* (22), and this observation might be due to the fact that the crRNA is incorporated into Cascade and that Cas6b is still bound to the crRNA thereby co-purifying with the FLAG-tagged Cas7. But although it copurifies with Cas5 and Cas7, it is not required to be part of the I-B Cascade for activity. Thus, the core part of the I-B Cascade seems to consist of Cas5, Cas7, and the crRNA. These results are confirmed by the observation that the 3′ handle can be completely removed. Thus, if the Cas6b protein is attached to Cascade via binding to the crRNA 3′ handle, this interaction is not essential.

##### Essential Parts of the 5′ Handle

Recent reports on the structure of the *E. coli* Cascade complex revealed that the first seven nucleotides of the crRNA 5′ handle form a hook that interacts with the Cas5, Cas7, and Cse1 proteins (the homologous protein in *Haloferax* would be the Cas8b protein) (42–44). Our data clearly show that in the *Haloferax* I-B system, the 5′ handle is also an important part of the crRNA. Only the first nucleotide of the 5′ handle can be mutated and deleted without loss of activity. This is in agreement with the *in vivo* situation where three different 5′ handles are generated (Fig. 1*A*.). In the structural analyses reported for the I-E Cascade complex, the first nucleotide of the 5′ handle interacts with Cas5 and Cas7 (42–44). In the *Haloferax* system, this interaction does not seem to be crucial for the activity. However, all other deletions in the 5′ handle abolished interference activity as follows: deletions of the first two nucleotides, of three internal nucleotides, and of all 5′ handle nucleotides yield a nonfunctional crRNA, confirming the importance of the 5′ handle.

##### Interaction of the crRNA 5′ Handle with the Protospacer Adjacent Motif

The nature of the last nucleotide of the 5′ handle (position −1) seems to be important; mutation of this nucleotide from C to G results in loss of activity, and only nucleotides C, A, and U are tolerated at this position. In *E. coli,* it has been shown that the −1 crRNA nucleotide is identical to the last PAM nucleotide and is derived from the invader (47–51), and thus the crRNA could base pair with the invader at this position (Fig. 3). It is not known whether the crRNA 5′ handle nucleotide (position −1) stems from the invader in the *Haloferax* I-B system. But the −1 crRNA nucleotide and invader complementary PAM nucleotide (in PAMs TTC and CAC, two of the six PAMs recognized by *Haloferax*) also have the potential to base pair. This base pair might be important for recognizing the correct target DNA sequence. The observation that the −1 nucleotide mutant C to U works but C to G does not work would confirm this hypothesis. However, the result that the C to A mutation is still active in interference does not fit. In addition, the complementary nucleotide of the other four PAMs recognized in the *Haloferax* system (TAT, TAA, TAG, and ACT) cannot base pair with the crRNA. In the I-E and I-F *E. coli* system, it has been shown that the interaction between the −1 crRNA nucleotide and the last complementary PAM nucleotide is not essential for invader recognition (52, 53). The recent structural data for the I-E Cascade complex confirm this earlier observation showing that in this system the −1 nucleotide of the crRNA is displaced by the Cas5 protein preventing interaction with the invader PAM sequence. The same displacement of the −1 nucleotide might happen in the *Haloferax* I-B Cascade. Also, the loss of activity of the C→G mutant could be explained by failure of the G to interact properly with the Cas5 protein.

In the I-E system, Cse1 (the homologous protein in *Haloferax* is Cas8b) interacts with the PAM sequence, and target recognition occurs via identification of the PAM sequence by the Cse1 protein (18, 53–55). The same might be true for the *Haloferax* I-B system, but the Cas8b protein should be able to identify six different PAMs as follows: TTC, CAC, TAT, TAA, TAG, and ACT. Taken together, our results suggest that a G at position −1 cannot interact properly with the Cas5 protein and that the *Haloferax* Cas8b would have to recognize all six different PAMs.

##### Essential Parts of the 3′ Handle

Mutational analysis of the icrRNA showed that the 3′ handle of the crRNA is completely dispensable. The shortest icrRNA found *in vivo* by RNAseq contained a five-nucleotide-long 3′ handle. According to the data reported here, this shorter crRNA version with only 49 nucleotides should also be active, because even an icrRNA with no 3′ handle is still active. Previous isolation of crRNAs from the *Haloferax* Cascade-like complex showed that the long and the short crRNA versions co-purify (22). It would be interesting to analyze whether only the short form is the active form and whether the long form has to be activated by 3′-processing to yield the short functional form. Currently, it is not known which enzyme(s) are catalyzing this further trimming of the crRNA 3′ end. As soon as this enzyme is identified, we could generate a strain that has the gene for the enzyme deleted and analyze whether the icrRNA with a long unprocessed 3′ handle is active.

A shortening of the crRNA 3′ handle has also been reported for the type I-B system of *Methanococcus maripaludis* and *Clostridium thermocellum* (56). Thus, it seems that in contrast to the I-A, I-E, and I-F systems, crRNAs of the I-B system are subjected to an additional 3′ trimming, as reported for the crRNAs in type III systems (14, 15).

##### Nature of the crRNA End Group Is Not Important

The pre-icrRNA is generated by the tRNA-processing enzymes to exactly the same product as the pre-crRNA generation by Cas6b. The only difference between the natural crRNA and the icrRNA is the nature of the 5′ and 3′ end groups. However, in the experiments reported here the nature of the end groups did not have any effect on the shortening of the icrRNA^66^ to icrRNA^49^ nor on the interference reaction. Taken together, the nature of the end groups seems not to be important for the interference reaction.

##### Minimal Type I-B crRNA

**FIGURE 4.**
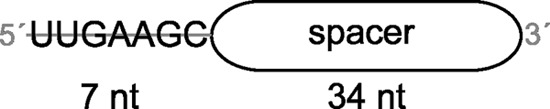
**Minimal crRNA.** The minimal crRNA for the defense reaction in *Haloferax* consists of a 7-nucleotide 5′ handle and a 34-nucleotide-long spacer.

Previously published data concerning the requirements for the spacer-protospacer interactions in the *Haloferax* I-B system showed that a 34-nucleotide-long spacer-protospacer interaction between crRNA and invader was sufficient (16). According to these published data and the results reported here, the minimal crRNA for the *Haloferax* type I-B system contains a 7-nucleotide-long 5′ handle, a 34-nucleotide-long spacer, and no 3′ handle (Fig. 4). Altogether, this crRNA would be 41 nucleotides long.

## Footnotes

4 The Cas proteins Cas6 and Cas8 are termed Cas6b and Cas8b in CRISPR-Cas type I-B.

5 A. Sabag-Daigle and C. J. Daniels, manuscript in preparation.

## Footnotes

3 The abbreviations used are:

CRISPR: clustered regularly interspaced short palindromic repeat
Cas: CRISPR associated
crRNA: CRISPR RNA
icrRNA: independently generated crRNA
nt: nucleotide
PAM: protospacer adjacent motif.

## References

[1] Marchfelder A. Fischer S. Brendel J. Stoll B. Maier L. K. Jäger D. Prasse D. Plagens A. Schmitz R. A. Randau L. (2012) Small RNAs for defence and regulation in archaea. Extremophiles 16, 685–696 22763819 10.1007/s00792-012-0469-5 PMC3432209

[2] Westra E. R. Swarts D. C. Staals R. H. Jore M. M. Brouns S. J. van der Oost J. (2012) The CRISPRs, they are A-Changin': how prokaryotes generate adaptive immunity. Annu. Rev. Genet. 46, 311–339 23145983 10.1146/annurev-genet-110711-155447

[3] Barrangou R. Marraffini L. A. (2014) CRISPR-Cas systems: prokaryotes upgrade to adaptive immunity. Mol. Cell 54, 234–244 24766887 10.1016/j.molcel.2014.03.011 PMC4025954

[4] Terns M. P. Terns R. M. (2011) CRISPR-based adaptive immune systems. Curr. Opin. Microbiol. 14, 321–327 21531607 10.1016/j.mib.2011.03.005 PMC3119747

[5] Bhaya D. Davison M. Barrangou R. (2011) CRISPR-Cas systems in bacteria and archaea: versatile small RNAs for adaptive defense and regulation. Annu. Rev. Genet. 45, 273–297 22060043 10.1146/annurev-genet-110410-132430

[6] Bikard D. Marraffini L. A. (2013) Control of gene expression by CRISPR-Cas systems. 1000Prime 10.12703/P5-47 PMC3816762 24273648

[7] Makarova K. S. Haft D. H. Barrangou R. Brouns S. J. Charpentier E. Horvath P. Moineau S. Mojica F. J. Wolf Y. I. Yakunin A. F. van der Oost J. Koonin E. V. (2011) Evolution and classification of the CRISPR-Cas systems. Nat. Rev. Microbiol. 9, 467–477 21552286 10.1038/nrmicro2577 PMC3380444

[8] Mojica F. J. Díez-Villaseñor C. García-Martínez J. Almendros C. (2009) Short motif sequences determine the targets of the prokaryotic CRISPR defence system. Microbiology 155, 733–740 19246744 10.1099/mic.0.023960-0

[9] Charpentier E. van der Oost J. White M. F. (2013) in CRISPR-Cas Systems (Barrangou R. van der Oost J., eds) pp. 115–144, Springer-Verlag, Berlin

[10] Chylinski K. Le Rhun A. Charpentier E. (2013) The tracrRNA and Cas9 families of type II CRISPR-Cas immunity systems. RNA Biol. 10, 726–737 23563642 10.4161/rna.24321 PMC3737331

[11] Vestergaard G. Garrett R. A. Shah S. A. (2014) CRISPR adaptive immune systems of Archaea. RNA Biol. 11, 156–167 24531374 10.4161/rna.27990 PMC3973734

[12] Reeks J. Naismith J. H. White M. F. (2013) CRISPR interference: a structural perspective. Biochem. J. 453, 155–166 23805973 10.1042/BJ20130316 PMC3727216

[13] Carte J. Wang R. Li H. Terns R. M. Terns M. P. (2008) Cas6 is an endoribonuclease that generates guide RNAs for invader defense in prokaryotes. Genes Dev. 22, 3489–3496 19141480 10.1101/gad.1742908 PMC2607076

[14] Hatoum-Aslan A. Maniv I. Marraffini L. A. (2011) Mature clustered, regularly interspaced, short palindromic repeats RNA (crRNA) length is measured by a ruler mechanism anchored at the precursor processing site. Proc. Natl. Acad. Sci. U.S.A. 108, 21218–21222 22160698 10.1073/pnas.1112832108 PMC3248500

[15] Hale C. R. Zhao P. Olson S. Duff M. O. Graveley B. R. Wells L. Terns R. M. Terns M. P. (2009) RNA-guided RNA cleavage by a CRISPR RNA-Cas protein complex. Cell 139, 945–956 19945378 10.1016/j.cell.2009.07.040 PMC2951265

[16] Maier L. K. Lange S. J. Stoll B. Haas K. A. Fischer S. Fischer E. Duchardt-Ferner E. Wöhnert J. Backofen R. Marchfelder A. (2013) Essential requirements for the detection and degradation of invaders by the *Haloferax volcanii* CRISPR/Cas system I-B. RNA Biol. 10, 865–874 23594992 10.4161/rna.24282 PMC3737343

[17] Semenova E. Jore M. M. Datsenko K. A. Semenova A. Westra E. R. Wanner B. van der Oost J. Brouns S. J. Severinov K. (2011) Interference by clustered regularly interspaced short palindromic repeat (CRISPR) RNA is governed by a seed sequence. Proc. Natl. Acad. Sci. U.S.A. 108, 10098–10103 21646539 10.1073/pnas.1104144108 PMC3121866

[18] Wiedenheft B. van Duijn E. Bultema J. B. Bultema J. Waghmare S. P. Waghmare S. Zhou K. Barendregt A. Westphal W. Heck A. J. Heck A. Boekema E. J. Boekema E. Dickman M. J. Dickman M. Doudna J. A. (2011) RNA-guided complex from a bacterial immune system enhances target recognition through seed sequence interactions. Proc. Natl. Acad. Sci. U.S.A. 108, 10092–10097 21536913 10.1073/pnas.1102716108 PMC3121849

[19] Künne T. Swarts D. C. Brouns S. J. (2014) Planting the seed: target recognition of short guide RNAs. Trends Microbiol. 22, 74–83 24440013 10.1016/j.tim.2013.12.003

[20] Maier L. K. Stoll B. Brendel J. Fischer S. Pfeiffer F. Dyall-Smith M. Marchfelder A. (2013) The ring of confidence: a haloarchaeal CRISPR/Cas system. Biochem. Soc Trans. 41, 374–378 23356314 10.1042/BST20120263

[21] Fischer S. Maier L. K. Stoll B. Brendel J. Fischer E. Pfeiffer F. Dyall-Smith M. Marchfelder A. (2012) An archaeal immune system can detect multiple protospacer adjacent motifs (PAMs) to target invader DNA. J. Biol. Chem. 287, 33351–33363 22767603 10.1074/jbc.M112.377002 PMC3460438

[22] Brendel J. Stoll B. Lange S. J. Sharma K. Lenz C. Stachler A. E. Maier L. K. Richter H. Nickel L. Schmitz R. A. Randau L. Allers T. Urlaub H. Backofen R. Marchfelder A. (2014) A complex of Cas proteins 5, 6, and 7 is required for the biogenesis and stability of crRNAs in *Haloferax volcanii*. J. Biol. Chem. 289, 7164–7177 24459147 10.1074/jbc.M113.508184 PMC3945376

[23] Hartmann R. K. Gössringer M. Späth B. Fischer S. Marchfelder A. (2009) The making of tRNAs and more–RNase P and tRNase Z. Prog. Mol. Biol. Transl. Sci. 85, 319–368 19215776 10.1016/S0079-6603(08)00808-8

[24] Allers T. Barak S. Liddell S. Wardell K. Mevarech M. (2010) Improved strains and plasmid vectors for conditional overexpression of His-tagged proteins in *Haloferax volcanii*. Appl. Environ. Microbiol. 76, 1759–1769 20097827 10.1128/AEM.02670-09 PMC2838008

[25] Miller J. H. (1972) Experiments in Molecular Genetics, Cold Spring Harbour Laboratory Press, Cold Spring Harbor, NY

[26] Delmas S. Shunburne L. Ngo H. P. Allers T. (2009) Mre11-Rad50 promotes rapid repair of DNA damage in the polyploid archaeon *Haloferax volcanii* by restraining homologous recombination. PLoS Genet. 5, e1000552 19593371 10.1371/journal.pgen.1000552 PMC2700283

[27] Allers T. Ngo H. P. Mevarech M. Lloyd R. G. (2004) Development of additional selectable markers for the halophilic archaeon *Haloferax volcanii* based on the leuB and trpA genes. Appl. Environ. Microbiol. 70, 943–953 14766575 10.1128/AEM.70.2.943-953.2004 PMC348920

[28] Cline S. W. Schalkwyk L. C. Doolittle W. F. (1989) Transformation of the archaebacterium *Halobacterium volcanii* with genomic DNA. J. Bacteriol. 171, 4987–4991 2768194 10.1128/jb.171.9.4987-4991.1989 PMC210307

[29] Fischer S. Benz J. Späth B. Maier L.-K. Straub J. Granzow M. Raabe M. Urlaub H. Hoffmann J. Brutschy B. Allers T. Soppa J. Marchfelder A. (2010) The archaeal Lsm protein binds to small RNAs. J. Biol. Chem. 285, 34429–34438 20826804 10.1074/jbc.M110.118950 PMC2966057

[30] Stoll B. (2013) Biology II, Ulm University, Ulm, Germany

[31] Norais C. Hawkins M. Hartman A. L. Eisen J. A. Myllykallio H. Allers T. (2007) Genetic and physical mapping of DNA replication origins in *Haloferax volcanii*. PLoS Genet. 3, e77 17511521 10.1371/journal.pgen.0030077 PMC1868953

[32] Gudbergsdottir S. Deng L. Chen Z. Jensen J. V. Jensen L. R. She Q. Garrett R. A. (2011) Dynamic properties of the *Sulfolobus* CRISPR/Cas and CRISPR/Cmr systems when challenged with vector-borne viral and plasmid genes and protospacers. Mol. Microbiol. 79, 35–49 21166892 10.1111/j.1365-2958.2010.07452.x PMC3025118

[33] Cock P. J. Fields C. J. Goto N. Heuer M. L. Rice P. M. (2010) The Sanger FASTQ file format for sequences with quality scores, and the Solexa/Illumina FASTQ variants. Nucleic Acids Res. 38, 1767–1771 20015970 10.1093/nar/gkp1137 PMC2847217

[34] Hoffmann S. Otto C. Kurtz S. Sharma C. M. Khaitovich P. Vogel J. Stadler P. F. Hackermüller J. (2009) Fast mapping of short sequences with mismatches, insertions and deletions using index structures. PLoS Comput. Biol. 5, e1000502 19750212 10.1371/journal.pcbi.1000502 PMC2730575

[35] Robinson J. T. Thorvaldsdóttir H. Winckler W. Guttman M. Lander E. S. Getz G. Mesirov J. P. (2011) Integrative genomics viewer. Nat. Biotechnol. 29, 24–26 21221095 10.1038/nbt.1754 PMC3346182

[36] Hölzle A. Fischer S. Heyer R. Schütz S. Zacharias M. Walther P. Allers T. Marchfelder A. (2008) Maturation of the 5S rRNA 5′ end is catalyzed *in vitro* by the endonuclease tRNase Z in the archaeon *H. volcanii*. RNA 14, 928–937 18369184 10.1261/rna.933208 PMC2327364

[37] Hölzle A. Stoll B. Schnattinger T. Schöning U. Tjaden B. Marchfelder A. (2012) tRNA-like elements in *Haloferax volcanii*. Biochimie 94, 940–946 22178322 10.1016/j.biochi.2011.12.002

[38] Gopalan V. Vioque A. Altman S. (2002) RNase P: variations and uses. J. Biol. Chem. 277, 6759–6762 11741968 10.1074/jbc.R100067200

[39] Schierling K. Rösch S. Rupprecht R. Schiffer S. Marchfelder A. (2002) tRNA 3′ end maturation in archaea has eukaryotic features: the RNase Z from *Haloferax volcanii*. J. Mol. Biol. 316, 895–902 11884130 10.1006/jmbi.2001.5395

[40] Garside E. L. Schellenberg M. J. Gesner E. M. Bonanno J. B. Sauder J. M. Burley S. K. Almo S. C. Mehta G. MacMillan A. M. (2012) Cas5d processes pre-crRNA and is a member of a larger family of CRISPR RNA endonucleases. RNA 18, 2020–2028 23006625 10.1261/rna.033100.112 PMC3479392

[41] Jore M. M. Lundgren M. van Duijn E. Bultema J. B. Westra E. R. Waghmare S. P. Wiedenheft B. Pul U. Wurm R. Wagner R. Beijer M. R. Barendregt A. Zhou K. Snijders A. P. Dickman M. J. Doudna J. A. Boekema E. J. Heck A. J. van der Oost J. Brouns S. J. (2011) Structural basis for CRISPR RNA-guided DNA recognition by Cascade. Nat. Struct. Mol. Biol. 18, 529–536 21460843 10.1038/nsmb.2019

[42] Jackson R. N. Golden S. M. van Erp P. B. Carter J. Westra E. R. Brouns S. J. van der Oost J. Terwilliger T. C. Read R. J. Wiedenheft B. (2014) Crystal structure of the CRISPR RNA-guided surveillance complex from *Escherichia coli*. Science 345, 1473–1479 25103409 10.1126/science.1256328 PMC4188430

[43] Mulepati S. Héroux A. Bailey S. (2014) Crystal structure of a CRISPR RNA-guided surveillance complex bound to a ssDNA target. Science 345, 1479–1484 25123481 10.1126/science.1256996 PMC4427192

[44] Zhao H. Sheng G. Wang J. Wang M. Bunkoczi G. Gong W. Wei Z. Wang Y. (2014) Crystal structure of the RNA-guided immune surveillance Cascade complex in *Escherichia coli*. Nature 515, 147–150 25118175 10.1038/nature13733

[45] Lintner N. G. Kerou M. Brumfield S. K. Graham S. Liu H. Naismith J. H. Sdano M. Peng N. She Q. Copié V. Young M. J. White M. F. Lawrence C. M. (2011) Structural and functional characterization of an archaeal clustered regularly interspaced short palindromic repeat (CRISPR)-associated complex for antiviral defense (CASCADE). J. Biol. Chem. 286, 21643–21656 21507944 10.1074/jbc.M111.238485 PMC3122221

[46] Brouns S. J. Jore M. M. Lundgren M. Westra E. R. Slijkhuis R. J. Snijders A. P. Dickman M. J. Makarova K. S. Koonin E. V. van der Oost J. (2008) Small CRISPR RNAs guide antiviral defense in prokaryotes. Science 321, 960–964 18703739 10.1126/science.1159689 PMC5898235

[47] Datsenko K. A. Pougach K. Tikhonov A. Wanner B. L. Severinov K. Semenova E. (2012) Molecular memory of prior infections activates the CRISPR/Cas adaptive bacterial immunity system. Nat. Commun. 3, 945 22781758 10.1038/ncomms1937

[48] Goren M. G. Yosef I. Auster O. Qimron U. (2012) Experimental definition of a clustered regularly interspaced short palindromic duplicon in *Escherichia coli*. J. Mol. Biol. 423, 14–16 22771574 10.1016/j.jmb.2012.06.037

[49] Swarts D. C. Mosterd C. van Passel M. W. Brouns S. J. (2012) CRISPR interference directs strand specific spacer acquisition. PLoS One 7, e35888 22558257 10.1371/journal.pone.0035888 PMC3338789

[50] Sinkunas T. Gasiunas G. Waghmare S. P. Dickman M. J. Barrangou R. Horvath P. Siksnys V. (2013) *In vitro* reconstitution of Cascade-mediated CRISPR immunity in *Streptococcus thermophilus*. EMBO J. 32, 385–394 23334296 10.1038/emboj.2012.352 PMC3567492

[51] Dupuis M. E. Moineau S. (2013) in CRISPR-Cas Systems (Barrangou R. van der Oost J., eds) pp. 171–200, Springer-Verlag, Berlin

[52] Almendros C. Guzmán N. M. Díez-Villaseñor C. García-Martínez J. Mojica F. J. (2012) Target motifs affecting natural immunity by a constitutive CRISPR-Cas system in *Escherichia coli*. PLoS One 7, e50797 23189210 10.1371/journal.pone.0050797 PMC3506596

[53] Westra E. R. Semenova E. Datsenko K. A. Jackson R. N. Wiedenheft B. Severinov K. Brouns S. J. (2013) Type I-E CRISPR-cas systems discriminate target from non-target DNA through base pairing-independent PAM recognition. PLoS Genet. 9, e1003742 24039596 10.1371/journal.pgen.1003742 PMC3764190

[54] Sashital D. G. Wiedenheft B. Doudna J. A. (2012) Mechanism of foreign DNA selection in a bacterial adaptive immune system. Mol. Cell 46, 606–615 22521690 10.1016/j.molcel.2012.03.020 PMC3397241

[55] Sternberg S. H. Haurwitz R. E. Doudna J. A. (2012) Mechanism of substrate selection by a highly specific CRISPR endoribonuclease. RNA 18, 661–672 22345129 10.1261/rna.030882.111 PMC3312554

[56] Richter H. Zoephel J. Schermuly J. Maticzka D. Backofen R. Randau L. (2012) Characterization of CRISPR RNA processing in *Clostridium thermocellum* and *Methanococcus maripaludis*. Nucleic Acids Res. 40, 9887–9896 22879377 10.1093/nar/gks737 PMC3479195

